# The prevalence and process of pediatric HIV disclosure: A population-based prospective cohort study in Zimbabwe

**DOI:** 10.1371/journal.pone.0215659

**Published:** 2019-05-22

**Authors:** Amy Finnegan, Lisa Langhaug, Katie Schenk, Eve S. Puffer, Simbarashe Rusakaniko, Yujung Choi, Simbarashe Mahaso, Eric P. Green

**Affiliations:** 1 Duke Global Health Institute, Durham, North Carolina, United States of America; 2 REPSSI, Belvedere, Harare, Zimbabwe; 3 George Mason University, College of Health and Human Services, Department of Global and Community Health, Fairfax, VA, United States of America; 4 University of Zimbabwe, Faculty of Medicine, Department of Community Medicine, Mount Pleasant, Harare, Zimbabwe; 5 Duke University, Department of Psychology and Neuroscience, Durham, NC, United States of America; 6 Batanai HIV & AIDS Service Organization, Masvingo CBD, Zimbabwe; The Ohio State University, UNITED STATES

## Abstract

**Introduction:**

The objective of this study was to estimate the prevalence of pediatric HIV disclosure in rural Zimbabwe and track the process of disclosure over time.

**Methods:**

We recruited a population-based sample of 372 caregivers of HIV-positive children ages 9 to 15 to participate in a survey about disclosure. Using data from this cross-sectional sample, we then identified a prospective cohort of 123 caregivers who said their HIV-positive child did not know his or her HIV status, and we followed this non-disclosed cohort of caregivers through two additional waves of data collection over the next 12 months. At each wave, we inquired about the timing and process of disclosure and psychosocial factors related to HIV disclosure.

**Results:**

The overall prevalence of disclosure in the cross-sectional sample was 66.9% (95% CI 62.0 to 71.5%). Only 26.9% of children knew how they were infected and that they can transmit the virus to others (i.e. “full disclosure”). Older children were more likely to know their status. Among the non-disclosed caregivers at baseline, nearly 60% of these children learned their HIV status over the course of the 12-month study period, but only 17.1% learned how they were infected and that they can transmit the virus to others. Most caregivers were satisfied with their child’s disclosure experience. Caregivers who had not disclosed their child’s HIV status to the child worried that disclosure would lead to stigma in the community, provoke questions from their child they would not be able to answer, or cause the child to reject the caregiver in anger.

**Conclusions:**

This study suggests that rates of pediatric HIV disclosure may be larger than typically reported, but also reinforces the idea that most children do not know key details about their illness, such as how they were infected and that they can infect others.

## Introduction

An estimated 2.1 million children are living with HIV [1]. Despite a compelling evidence-base for the benefits of developmentally appropriate pediatric HIV disclosure, many of these children are unaware of their condition, do not understand the importance of remaining adherent to antiretroviral therapy, and enter adolescence without the knowledge that they could pass the infection to others. The American Academy of Pediatrics and the World Health Organization recommend that HIV-positive children should be informed of their serostatus [2–4] when they reach school-age, but disclosure is not an easy process for parents and guardians and many keep it a secret or reveal limited details. Caregivers who avoid disclosure often believe that their child is too young to understand or that disclosure will cause psychological harm, prompt the child to ask difficult questions, or share this new information with others, potentially exposing the child and family to stigma [5–10]. Yet, research suggests that there is more to be gained from appropriate disclosure than risked by secrecy. Children who know their status have been found to exhibit higher self-esteem, fewer behavior problems, and less psychological distress—including fewer symptoms of depression and anxiety—compared to their non-disclosed peers [11–16]. After disclosure, they may also have improved social functioning and more social support, positive attitudes about their health, and greater hope for the future [6, 7, 12, 16, 17].

Studies suggest that the rate of disclosure is as low as 1.7% [18] and as high as 75% [19], but the narrative of pediatric HIV disclosure in the literature is that rates are low [20–23]. The true rate may indeed vary widely by country, setting (urban/rural), context (HIV prevalence), but it is possible that the wide spread of more than 70 percentage points is driven in part by methodological differences in study design. It is also possible that disclosure rates are not as low as typically assumed.

For instance, child age is a fairly consistent predictor of disclosure status across the literature [20], yet few studies of disclosure disaggregate rates of disclosure by age. Therefore, reported point estimates of disclosure mask the underlying variability by age. Additionally, most studies estimate the rate of disclosure using small convenience samples that do not attempt to be representative of the target population under investigation. A particular limitation is that most studies only recruit from a small number of clinics. To the extent that disclosure rates vary by clinic, the decision to purposively select a handful of clinics for recruitment can result in biased estimates of disclosure.

The literature recognizes the importance of understanding disclosure as a ‘process’ that unfolds over time [24, 25], but the conceptualization of this process is informed largely by cross-sectional, qualitative studies that are rich in detail, but may be low in generalizability. Missing are prospective, longitudinal studies that examine the process of disclosure over time [26]. Moreover, studies seldom report results by different definitions of disclosure (e.g., full, partial); most studies only report the percentage of children who know that the name of their condition is HIV without reporting how many know how they were infected and that they can spread the virus to others.

This study attempts to address these limitations by recruiting a population-based sample of caregivers of HIV-positive children, estimating the prevalence of disclosure to children among the cross-sectional sample, and then following the caregivers of the non-disclosed children over the next 12 months to study the process of disclosure. We anchor these results in the broader literature on pediatric disclosure.

## Methods

### Research design

We recruited a population-based sample of caregivers of HIV-positive children ages 9 to 15 years to estimate the prevalence of pediatric HIV disclosure. After this baseline wave of data collection, we followed a prospective cohort of caregivers from the sample whose children were unaware of their HIV-positive status to understand the process of disclosure.

### Setting and participants

The target population for this study was primary caregivers—parents and guardians—of HIV-positive children ages 9 to 15 living in rural Zimbabwe. The accessible population was limited to the subset of these caregivers living in Bikita and Zaka districts in Masvingo Province whose HIV-positive children were receiving antiretroviral therapy (or were in pre-ART). We selected this location because our partner agency had established relationships with 42 of the 48 HIV care clinics in these districts and supported a wide network of Community HIV & AIDS Support Agents (CHASAs) deemed essential for participant recruitment and retention.

Masvingo Province is home to approximately 1.5 million people, 23% of whom live in Bikita and Zaka districts [27]. The demographics of Masvingo are similar to the rural clusters in the 2015 Zimbabwe Demographic and Health Survey (DHS) [28], suggesting that the accessible population from which we sampled may be representative of the target population of rural caregivers. As of 2015, the adult HIV prevalence rate in Masvingo was estimated to be 12.9%; the prevalence of HIV among children aged 0 to 14 years was 1.5% [28].

### Procedures

#### Cross-sectional study of pediatric HIV disclosure prevalence

At the time of this study, it was estimated that nearly 8 out of 10 HIV-positive children in Masvingo were enrolled in ART [29]. This made clinic-based recruitment a valid strategy for obtaining a representative sample of caregivers of HIV-positive children.

**Fig 1:**
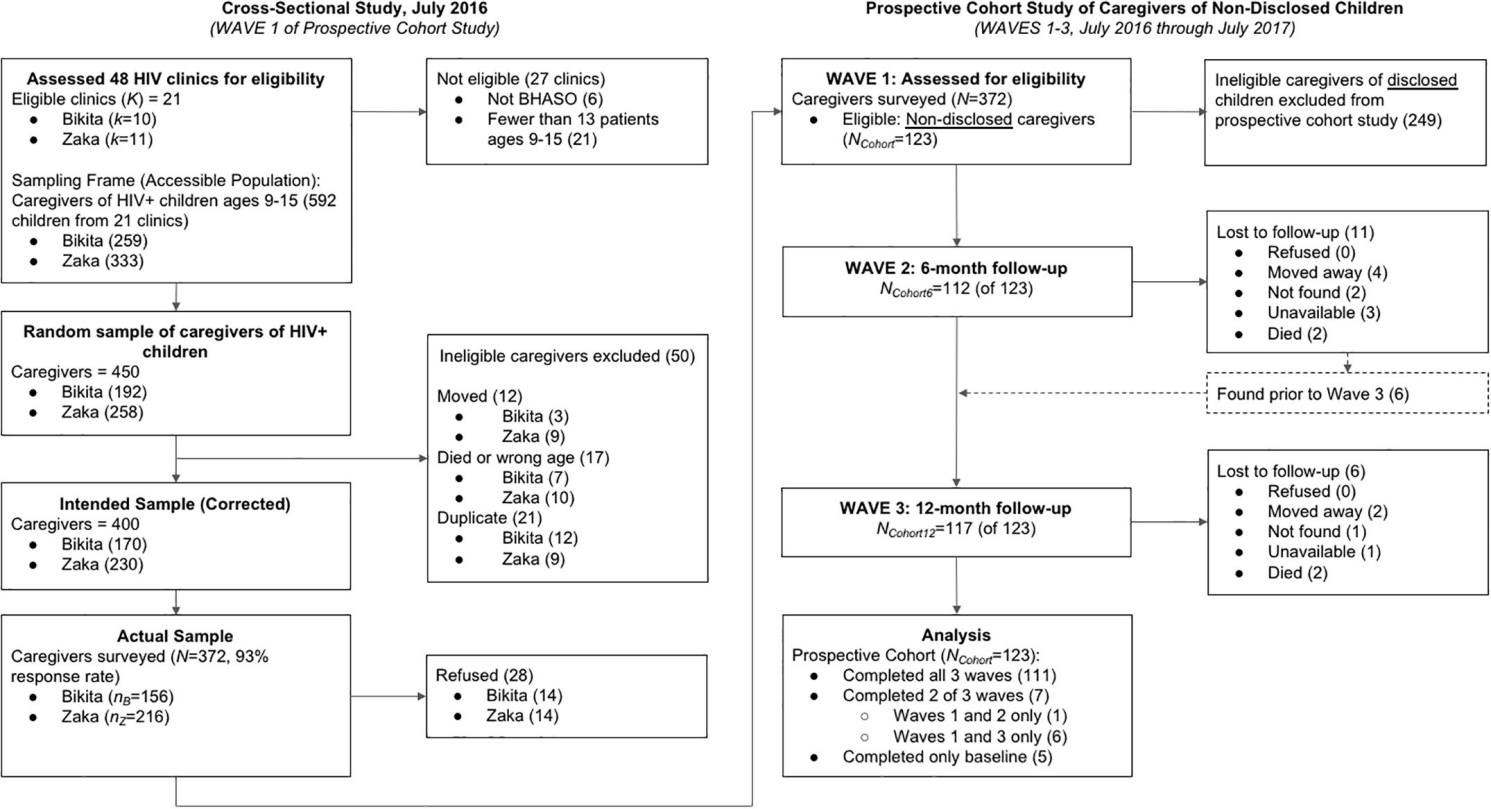
Participant flow diagram.

We constructed a sampling frame of pediatric HIV patients receiving ART or pre-ART services at 42 of the 48 HIV clinics in Bikita and Zaka Districts that were part of our partner network, the Batani HIV and AIDS Service Organization (BHASO). 3.7% of children in the sampling frame were on pre-ART vs. 96.3% on ART. Once we had an initial list of patient load by clinic, we excluded an additional 21 facilities that provided HIV care to fewer than 13 patients ages 9 to 15 for logistical reasons (see Figs A.1 and A.2 in S1 Appendix for an analysis of these exclusions). From the remaining 21 clinics, we used ART and pre-ART registers to develop a sampling frame of 513 eligible children. We then used stratified simple random sampling by district (proportional to size) to select a random sample of 450 children; the target sample size was driven by the needs of a larger study on disclosure readiness. CHASAs (Community HIV and AIDS Support Agents employed by BHASO) assigned to each clinic updated this sample through the removal of children no longer living in the district, children who had died, and duplicate records. After these corrections, the intended sample consisted of 400 children (Fig 1).

Between June and August 2016, our team of Zimbabwean enumerators attempted to enroll and survey caregivers in the intended sample. Overall, the team surveyed 372 of 400 caregivers, a response rate of 93.0%. All surveys were conducted individually in a private setting at the clinics where caregivers pick up medications for their children. Enumerators read each survey item aloud in Shona and recorded participants’ responses on a tablet [30]. Healthcare workers were not involved in the study or involved with the surveys.

#### Prospective cohort study of the disclosure process

Using data from the first wave of data collection, we identified a cohort of 123 caregivers who said their HIV-positive child did not know his or her HIV status, and we followed this “non-disclosed” cohort through two additional waves of data collection over the next 12 months. We used the procedures described above and attempted to survey all 123 caregivers in this cohort at 6-months and 12-months following the first wave of data collection.

### Measures

See the study repository for a copy of the study materials described below [31]. Survey items were developed through a literature review, focus group discussions with 17 HIV-positive adolescents, 18 parents of HIV-positive children, and health workers at 23 clinics using free-listing and card sorting activities, and cognitive interviewing with a separate group of caregivers.

#### Caregiver and child demographics

We used relevant modules from the 2015 Zimbabwe DHS questionnaire to collect demographic data on participants, their children, and their households [28].

#### Caregiver-reported pediatric HIV disclosure

We asked a series of questions to assess what the caregiver believes the child knows about his or her HIV status and condition, when the child learned this information, and from whom. We also asked whether the caregiver wanted the child to learn this information when they did. Specifically, we probed whether the child knows: (a) that he or she has a chronic condition requiring medication; (b) that he or she has a medical condition called HIV; (c) how he or she was infected with HIV; and (d) that he or she can spread the virus to others. “Partial disclosure” in this study refers to knowing (b), (c), *or* (d), but not all three. “Full disclosure” refers to knowing (b), (c), *and* (d) [7, 32, 33]. In order to be consistent with other studies of pediatric HIV disclosure, we use the term “disclosed” throughout to refer to children who know they have a virus called HIV (b).

For caregivers of disclosed children, we asked whether they found out their child’s HIV status before the child, on the same day as the child, or after the child. For caregivers of non-disclosed children at Wave 1, we asked about their intentions to disclose their child’s HIV status within the next 12 months. We also asked these caregivers (yes or no) whether they had begun to assess their child’s readiness for disclosure, taken any steps to prepare their child, consulted with a health care worker about disclosure, or made a plan to disclose.

#### Caregiver-reported worries about disclosure

We created a series of 15 items to assess caregivers’ worries about disclosing the child’s status to the child. For instance, “I worry that telling [him/her] will make [him/her] too sad.” Caregivers responded to each item on a 4-point scale from (0) ‘not at all worried’ to (3) ‘very worried’.

In the final wave of the prospective cohort, we trained the enumerators in active listening and encouraged them to take notes about the stories caregivers told them during the process of data collection. At the end of each day, a trained interviewer collected these stories from the enumerators.

### Data analysis

We conducted our analysis with R [34]. Using Wave 1 data from the cross-sectional sample, we estimated the prevalence of disclosure with 95% confidence intervals. For the prospective cohort analysis, we calculated the cumulative incidence of disclosure over the follow-up period [35]. We explored the disclosure process in both samples through a descriptive analysis. Caregiver disclosure stories were coded and examined thematically by two members of the study team to add illustrative detail to survey findings.

### Ethical review

The study protocol was approved by the Joint Parirenyatwa Hospital and College of Health Sciences Research Committee, the Medical Research Council of Zimbabwe, and Institutional Review Boards at Duke University and George Mason University. All study participants provided written informed consent.

## Results

### Sample characteristics

372 of 400 eligible caregivers in the intended sample enrolled in the cross-sectional study and completed the Wave 1 survey (93.0% response rate). According to caregiver reports, 123 children (33.1%) did not know their HIV status at baseline. We followed this cohort of 123 caregivers through two additional rounds of data collection. We successfully surveyed 91.1% in Wave 2 and 95.1% in Wave 3.

Table 1 reports the demographics of the sample at baseline and univariate odds ratios of disclosure at baseline by demographic characteristics of children and caregivers. Most caregivers in the cross-sectional survey sample (Wave 1) were females, and the average age was 47.8 years. Almost half of caregivers were biological parents and about one-third were grandparents. (Descriptive results for the remainder are available in Table B.1 in S1 Appendix) About two-thirds had completed primary education or more, and half were married. Two-thirds of caregivers disclosed to the survey team that they themselves were HIV-positive. The average age of HIV-positive children linked to these caregivers was 12.1 years, and about half were female.

**Table 1:** Characteristics of the cross-sectional sample and prospective cohort at Wave 1.

Variable	Cross-Sectional Sample	Prospective Cohort		Odds of disclosure at baseline (95% CI)
		Excluded (Disclosed)	Included (Non-Disclosed)	
Caregivers				
N (%)	372 (100.0)	249 (66.9)	123 (33.1)	
Female (%)	87.6	86.7	89.4	0.77 (0.38, 1.50)
Mean Age (SD)	47.8 (12.9)	47.1 (12.1)	49.1 (14.5)	0.99 (0.97, 1.00)
Biological Caregiver (%)	45.4	48.6	39.0	1.48 (0.95, 2.30)
Grandparent Caregiver (%)	29.0	25.7	35.8	0.62 (0.39, 0.99)
Completed Primary (%)	61.8	63.5	58.5	1.23 (0.79, 1.91)
Married (%)	53.2	49.0	61.8	0.59 (0.38, 0.92)
HIV + (%)	62.1	65.1	56.1	1.46 (0.94, 2.27)
Poorest 2 Wealth Quintiles (%)	66.4	66.7	65.9	1.04 (0.65, 1.63)
Reference children				
Female (%)	51.1	50.6	52.0	0.94 (0.61, 1.46)
Mean Age (SD)	12.1 (1.8)	12.3 (1.7)	11.7 (1.8)	1.23 (1.09, 1.40)
Completed Primary (%)	21.8	27.3	10.6	3.18 (1.73, 6.26)

Odds ratios (OR) and 95% confidence intervals of the odds of disclosure at baseline by each demographic characteristic also measured at baseline. Variables summarized as percentages (%) are indicators coded yes = 1/no = 0 in which the OR represents the association between the characteristic (Married: the caregiver is married) and reporting that the child knows his or her HIV status. The age variables are summarized as means and standard deviations, and the OR represents the association between an increase in age of 1 year and the parent’s report that the child knows his or her HIV status.

### Cross-sectional results, Wave 1

#### Prevalence of disclosure

The overall prevalence of disclosure in the cross-sectional sample was 66.9% (95% CI 62.0 to 71.5%; see Table B.2 in S1 Appendix). Disclosure rates were similar between districts: 64.1% in Bikita and 69.0% in Zaka. Across the 21 study clinics, disclosure rates ranged from 47.8 to 85.7%; clinic size was not associated with disclosure rates (see Fig B.1 in S1 Appendix). Among the children who knew that they were HIV-positive, 69.9% knew how they were infected and 48.6% knew that they could pass the infection to someone else. 26.9% of children were fully disclosed to at baseline (see Table B.2 in S1 Appendix).

#### Age of disclosure

**Fig 2:**
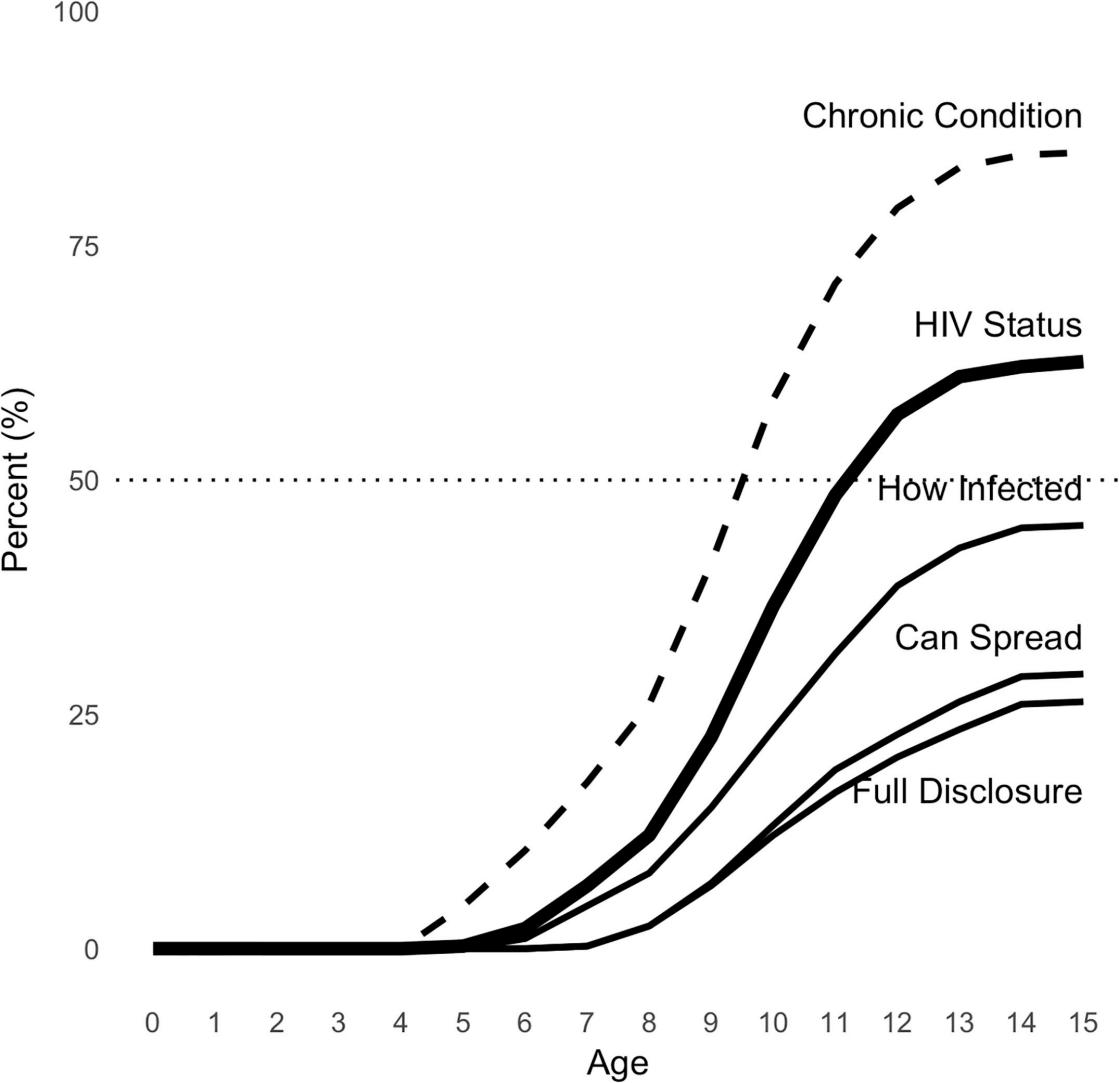
Cumulative distribution of knowledge of HIV status by age. Each line indicates the percent of children at each age who know facts about their HIV status. The dotted line indicates 50% of children in the sample knowing their status. Sample includes all 372 children whose caregivers were surveyed as part of the cross-sectional survey (Wave 1).

As shown in Fig 2, disclosure is a process that unfolds over time rather than a one-time event. This figure shows the cumulative distribution of children’s knowledge about their condition by age. In most cases, children first learn that they have a chronic health condition, and then learn the name HIV, how they were infected, and that they can transmit the virus to others. In this sample, some caregivers reported that children learned they had a chronic health condition as early as age 4, though this was rare. Children began learning they were infected with HIV as early as age 5, but most did not know this until age 11. The rate of knowing one’s HIV status increases quickly between ages 7 and 12 and flattens out at ages 13 to 15. As shown in Table 1, older children are more likely to know their status. See Fig B.2 in S1 Appendix for the predicted probability of disclosure of HIV status by age.

#### Who does the disclosing

Most disclosed caregivers in the cross-sectional sample reported learning about their child’s HIV status before the child (150/249; 60.2%). Among these caregivers (*N* = 150), 76.0% reported being the one to disclose to the child. 32.7% of the caregivers who told the child themselves (*N* = 114) reported taking steps to prepare the child to receive the information. In particular, 60.9% sought help in preparation from health workers and 20.9% turned to family members or friends. Most of the caregivers who made the disclosure on their own (*N* = 114) said they viewed disclosure as a process (71.8%) and did not pick a specific day to disclose to the child (75.5%). The majority of these caregivers felt that the disclosure experience was ‘somewhat’ or ‘very’ good for them (96.4%) and the child (95.1%).

Among the caregivers who found out their child’s HIV status before the child but did not disclose to the child themselves (*N* = 36), 61.1% said that the child learned their status from a health worker, and 38.9% said the child learned from someone else like another family member. Most or all of these caregivers confirmed that they wanted the disclosure to happen when it did (81.8%) and said that the disclosure experience was ‘somewhat’ or ‘very’ good for them (100.0%) and the child (92.9%).

While most caregivers learned about the child’s status before the child, 30.1% (75/249) learned on the same day as the child and 9.6% (24/249) learned after the child. Children of caregivers who learned the same day as the child were 12.5 years old, on average, and about half were cared for by biological caregivers (53.3%). In roughly half the cases where a caregiver learned on the same day as the child (56.0%), it was because a health worker disclosed to the child and their caregiver when the test results were available. Among this subset of health worker disclosures (*N* = 42, 35 responding), every caregiver reported that they wanted the child to learn when they did, and 86.1% reported that they perceived the child’s disclosure experience to be ‘somewhat’ or ‘very’ good.

### Longitudinal results from the prospective cohort

We followed the 123 non-disclosed caregivers for a year to learn how the disclosure process unfolded over time. Just over one third of these caregivers endorsed the idea that their child *should* know their status, and a slightly smaller percentage said they intended to disclose this information to the child within 12 months (see Table 2). Caregivers’ behavioral intentions differed by child’s age at baseline; caregivers of older children were twice as likely as caregivers of younger children to report an intention to disclose within the year.

**Table 2:** Behavioral intentions to disclose and follow-through among the prospective cohort.

	Child Age		Total
	less than 12 yrs	12+ years	
N	52	61	113
Child should know HIV status (%, Wave 1)	31.4	41.0	37.1
Caregiver intends to tell child within 12 months (%, Wave 1)	23.1	41.0	32.7
Child knows HIV status at 12 months (%, Wave 3)	51.9	63.5	59.6
Among caregivers who intended to disclose, N:	12	25	37
Child knows HIV status at 12 months (%)	81.8	88.0	83.8
a. Assessed child’s readiness (%)	66.7	68.0	67.6
b. Took steps to prepare child (%)	41.7	60.0	54.1
c. Consulted health care worker (%)	58.3	48.0	51.4
d. Made a plan to disclose (%)	91.7	76.0	81.1
None of a-d (%)	0.0	4.0	2.7
Any of a-d (%)	100.0	96.0	97.3
All of a-d (%)	0.0	20.0	13.5

Note. Child age refers to age at baseline. Sample includes 113 caregivers in the prospective cohort who responded to the question about intention to disclose their child’s HIV-positive status to the child within 12 months from baseline and who were surveyed at 12 months. Steps a-d are indicators of preparation at Waves 1, 2 or 3.

**Fig 3:**
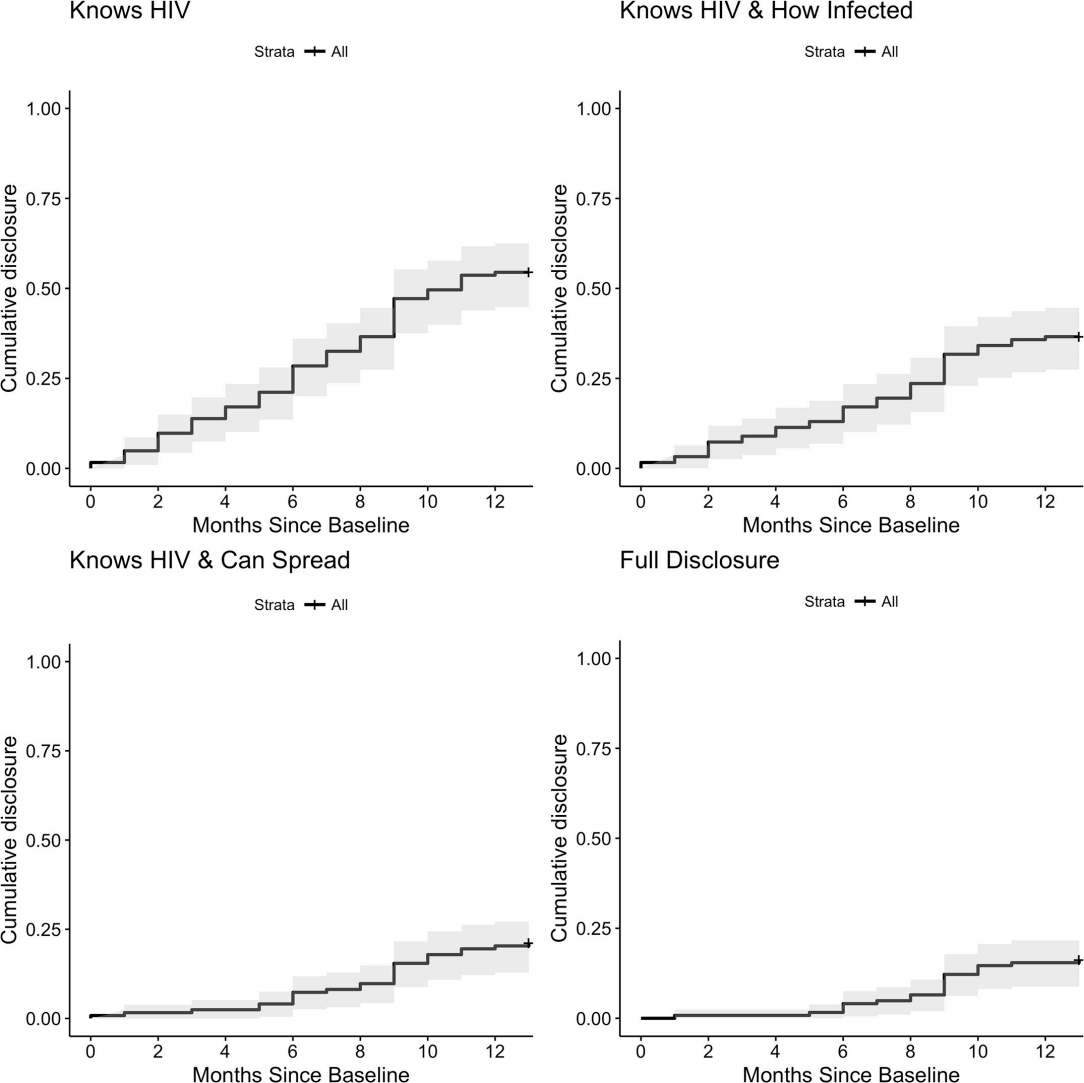
Rates of learning facts about HIV status among the prospective cohort. This figure shows the cumulative incidence of disclosure to children in the prospective cohort. Each period represents 1 month from the baseline survey (Wave 1).

Over the course of the next year, nearly 60% of children in the prospective cohort learned their status, but only 17.1% also learned how they were infected and that they can transmit the virus to others (i.e., full disclosure). Fig 3 shows the cumulative incidence of disclosure over this prospective period. As also observed in the cross-sectional study, the general progression of disclosure among the prospective cohort was for the child to first learn that the name of the disease is HIV, then how he or she was infected, and finally that he or she can infect others (see Fig B.3 in S1 Appendix for an illustration of the pathways of disclosure, see Fig B.4 in S1 Appendix for disclosure rates disaggregated by age, and see Table B.2 in S1 Appendix for disclosure rates across waves).

Comparing the data on disclosure status at 12-months to disclosure intentions expressed at baseline, there appears to be a clear link between behavioral intention and actual behavior. Caregivers who intended to disclose to the child were almost twice as likely to act compared to caregivers who did not intend to disclose (see Table 2). Almost all caregivers who intended to disclose took some preparatory action within the year even if they did not begin disclosure. Some caregivers said the surveys prompted them to think about disclosing to their child, but we did not observe a large spike in disclosure following the first two survey waves (see Fig B.5 in S1 Appendix).

Among the 49 caregivers who did not disclose within the year, several key barriers to disclosure emerged. First, more than two-thirds of these caregivers (67.3%) agreed that their child was too young to learn their status; the mean child age at Wave 3 among non-disclosed caregivers was 12.6 years. A similar percentage (69.4%) expressed a belief that their child was not strong enough to handle the disclosure, and 45.7% said they were worried that telling the child would be harmful. Grandmothers in particular spoke about the fragility of their grandchildren who they felt were still struggling with a parent’s death. One grandmother worried that if her grandson learns that he is suffering from the same disease that killed his mother, ‘he might be shocked and die’.

Nearly half of non-disclosed caregivers (47.9%) expressed a worry that disclosure would lead the child to experience stigma in the community. One mother shared her observations of other children refusing to play with HIV-positive children. A father spoke about seeing another child be teased about his HIV condition only to die soon after, leading the father to worry that his child might suffer the same fate.

Just over half of these caregivers (55.3%) said they worried that disclosure would lead the child to ask more tough questions, and 46.9% said they would not be able to provide answers. In some of these cases, these caregivers expressed a desire for the nurse to disclose to the child, feeling that they could provide a more detailed explanation as health professionals. See Table B.3 in S1 Appendix for a complete list of the concerns non-disclosed caregivers endorsed at Wave 3.

## Discussion

This study is among the first to draw a population-based sample of caregivers of children receiving ART or pre-ART at HIV clinics, estimate the prevalence of pediatric disclosure as reported by caregivers, and track the process of disclosure over time among the non-disclosed caregivers. Based on a cross-sectional sample of 372 caregivers of children and adolescents aged 9 to 15, we estimated that the overall prevalence of disclosure—that is, the caregiver reported that the child knows he or she has a virus called HIV—was 66.9%.

**Fig 4:**
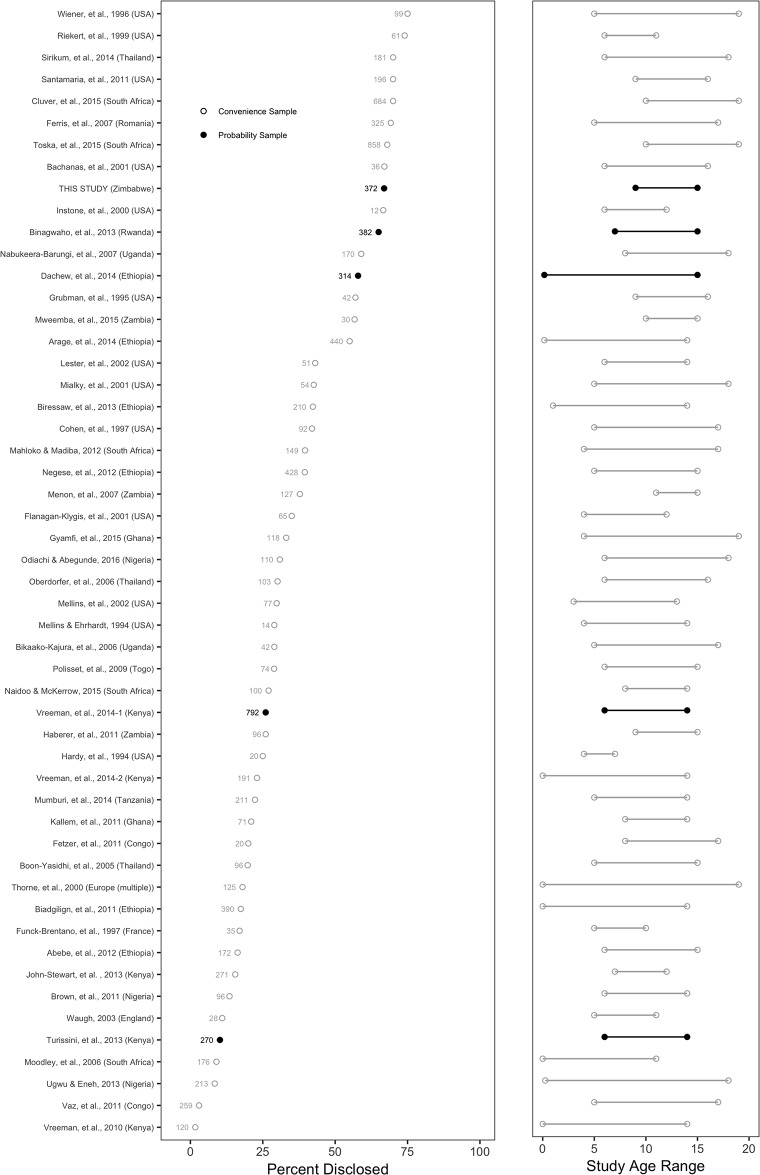
Published rates of HIV disclosure to children. This figure displays the rates of pediatric HIV disclosure (i.e. the child knows that the name of the illness they have is HIV) reported in 52 studies (including this study), the location and sample size of each study, whether the study used probability sampling methods (black filled dots), and the age range of the children. We compiled the data for this figure from recent systematic reviews [4, 7, 20–23, 44] and our own supplemental search of the literature. See Appendix C in S1 Appendix for additional details.

Fig 4 places this disclosure finding in the broader context of the published literature on pediatric HIV disclosure, which is characterized by diversity in methods and findings (see Appendix C in S1 Appendix for a description of our approach). Published disclosure rates using this same definition of knowing one has a virus called HIV range from nearly 0% to 75%, sample sizes range from 12 to 858, and child age ranges from less than 1 year to 19 years. The prevalence we observed in the current study, 66.9%, falls in the top 20% among all available estimates. Most other study results, however, are based on data from small, non-probability samples, which complicates the estimation and interpretation of “prevalence.”

In terms of sample size, sampling method, and child age range, our study design is most comparable to Binagwaho et al. (Rwanda, *N = 382*, 65%) [36], Vreeman et al. (Kenya, *N = 792*, 26%) [37], and Turissini et al. (Kenya, *N = 270*, 11.1%) [38]. It is not possible to determine with any certainty why these studies found such different rates of disclosure, but differences in methodology could provide a partial explanation. Our study and that of Binagwaho et al. [36]—which found very similar rates of disclosure, 66.9% and 65%, respectively—both recruited population-based samples. In comparison, Turissini et al. [38] only sampled from one large referral clinic in a major urban area and Vreeman et al. [37] sampled from only four clinics chosen purposively. To the extent that disclosure rates vary widely across clinics, limited and purposive selection of clinics could bias the results. In our study, we estimated that disclosure rates ranged from 47.8 to 85.7% across the 21 clinics in the study. For these reasons, and in light of the similarities in approach and findings with Binagwaho et al. (2013), we interpret our collective results to suggest that the prevalence of disclosure to children might be higher than typically reported.

Knowing the name of the virus is only part of the picture, of course. The World Health Organization recommends beginning the disclosure process at age 6 and disclosing HIV status fully by age 12 [39]. However, our data suggest that by age 12, only slightly more than half of children knew their status, and about 1 in 3 knew that they could infect others. Overall, only 26.9% of children in the cross-sectional survey met the criteria for full disclosure, according to caregiver report. This is comparable to a cross-sectional study in Uganda of children ages 5 to 17 years that used the same definition and reported a full disclosure rate of 31% [33].

Another contribution of this study is that we followed the cohort of non-disclosed caregivers prospectively, giving us the opportunity to learn about the process of disclosure over the course of a year. The only other prospective cohort study of pediatric HIV disclosure was conducted in the United States and Puerto Rico and focused on the consequences of disclosure [40]. In focusing on the process, we found that there are various pathways to full disclosure but that children generally tend to learn information over time with knowledge of potential forward transmission coming last. We also found that caregivers who reported an intention to disclose within the next year were more likely to actually disclose during this period, consistent with the theory of planned behavior and reasoned action approach [41, 42]. Future prospective studies would be valuable for understanding similarities and differences in how these processes unfold across varied contexts and cultures.

One limitation of this study, and others in this field, is our reliance on caregiver report of the child’s disclosure status. While focusing on caregivers helped us to avoid situations of accidental disclosure that are more likely when interviewing children, we lack awareness of what disclosed children really understand about their condition [43]. Our estimates may also be biased since we relied on caregiver report; bias could work to underestimate or overestimate disclosure.

Another limitation is that, for logistical reasons, we excluded 21 of 42 facilities that provided HIV care to fewer than 13 patients ages 9 to 15 years. We did not have the resources to visit clinics and survey only 1 or 2 caregivers who might have been randomly selected from these small clinics. One concern about excluding the smallest facilities was that they were systematically different from the included facilities in terms of HIV prevalence, which might be associated with rates of disclosure. In Figs A.1 and A.1 in S1 Appendix we demonstrate why this is unlikely. We find no relationship between HIV prevalence and clinic size, nor between pediatric HIV disclosure rates and HIV prevalence.

Finally, we note that while sampling from a population of children on ART and pre-ART is a valid strategy in this context given that most HIV positive children are on ART, it is possible that the results may not generalize to settings where pediatric ART coverage is low. It seems logical to assume that disclosure rates may be lower in such settings. Also, while we demonstrate that the provinces included in this study are similar in some ways to other rural provinces in Zimbabwe based on our review of DHS data, it is possible that the results we obtained from our accessible population do not generalize to the target population because of unmeasured differences in related cultural or contextual factors.

### Conclusions

This study suggests that rates of pediatric HIV disclosure may be higher than typically reported, but also reinforces the idea that most children do not know key details about their illness, such as how they were infected and that they can infect others. Our results support the idea that HIV disclosure is a process that unfolds over time. Nurses and medical providers can be a trusted source of support during the disclosure process and can play an important role by addressing common barriers, such as misunderstandings about developmentally appropriate timeframes for disclosure and concerns that children are not resilient enough to handle the disclosure process.

## Supporting information

S1 Appendix (PDF)
