# Supplementary material for: The prevalence and process of pediatric HIV disclosure: A population-based prospective cohort study in Zimbabwe
Source: PLoS One. 2019 May 22;14(5):e0215659. doi: 10.1371/journal.pone.0215659 (PMC6530961; doi:10.1371/journal.pone.0215659)
Supplement: S1 Appendix — (PDF) [file pone.0215659.s001.pdf]

Appendices

A Analysis of Clinic Eligibility Criteria

We constructed a sampling frame of pediatric HIV patients receiving ART or pre-ART services at 42 of the 48 HIV care clinics in Bikita and Zaka Districts; the 6 excluded facilities were not part of the BHASO network. Once we had an initial list of patient load by clinic, we excluded an additional 21 facilities that provided HIV care to fewer than 13 patients ages 9 to 15.

One concern about excluding these smaller facilities was that they were systematically different from the included facilities in terms of HIV prevalence, which might be associated with rates of disclosure. To examine this possibility, we compiled GPS coordinates of all included and excluded facilities and plotted clinics on a map of interpolated estimates of HIV prevalence among those aged 15 to 49 as measured in the 2010 Zimbabwe Demographic and Health Survey (ZDHS) [1, 2].

Figure A.1 shows that there is no relationship ($p < 0.22$) between HIV prevalence of 15 to 49 year olds and the number of children between ages 9-15 on ART or pre-ART at all clinics in our sampling frame. The black points are clinics that were selected (clinic size is greater than or equal to 13). The grey points are excluded clinics. Additional analysis confirms that there is no relationship between the residuals in a regression of HIV prevalence at each clinic on clinic size (results not shown).

We also examined whether our estimated disclosure rates per clinic are related to estimated HIV prevalence in the area. Figure A.2 shows that among clinics in our survey, there is no relationship ($p < 0.49$) between HIV prevalence and the percent of children who know their HIV status. Additional analysis confirms that there is no relationship between the residuals in a regression of HIV prevalence at each clinic on clinic-level HIV disclosure (results not shown).

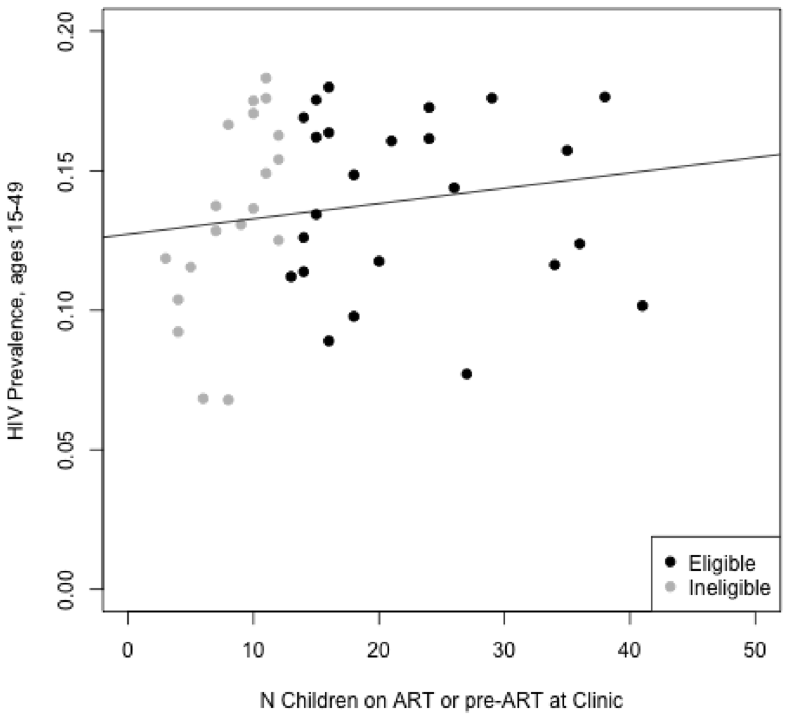

Figure A.1: **Prevalence of HIV vs. clinic size.** Each dot represents a clinic. Black points represent included clinics, and grey points represent excluded clinics. There is no relationship between the prevalence of HIV and clinic size.

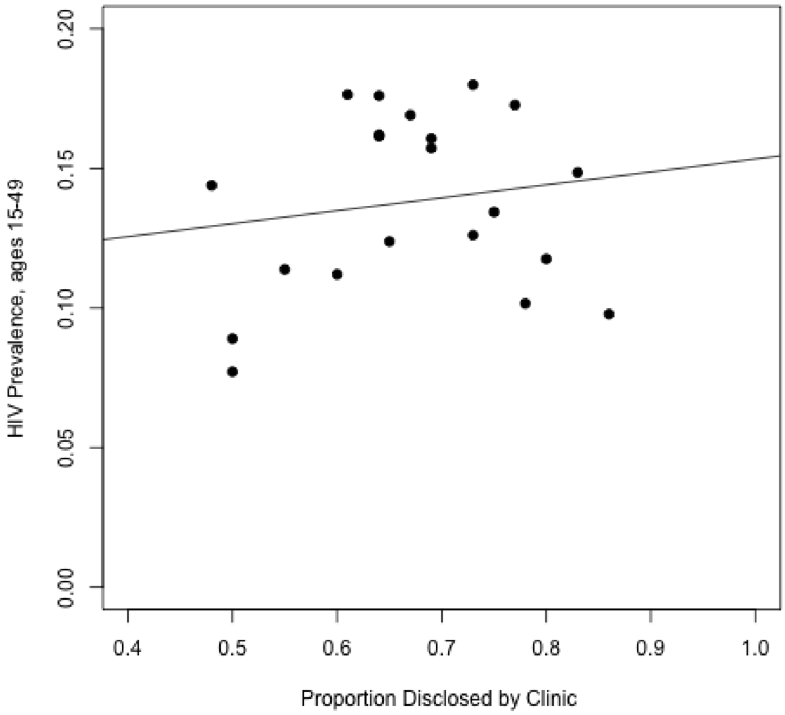

Figure A.2: **Prevalence of HIV vs. prevalence of pediatric HIV disclosure.** This figure shows the prevalence of HIV (ages 15-49) around each study clinic and the percentage of children who know their HIV status at each clinic. There is no relationship between HIV prevalence and disclosure.

Based on this exercise, we concluded that excluding clinics with fewer than 13 children on ART or pre-ART did not systematically alter our estimates of HIV disclosure.

B Supplemental Tables and Figures

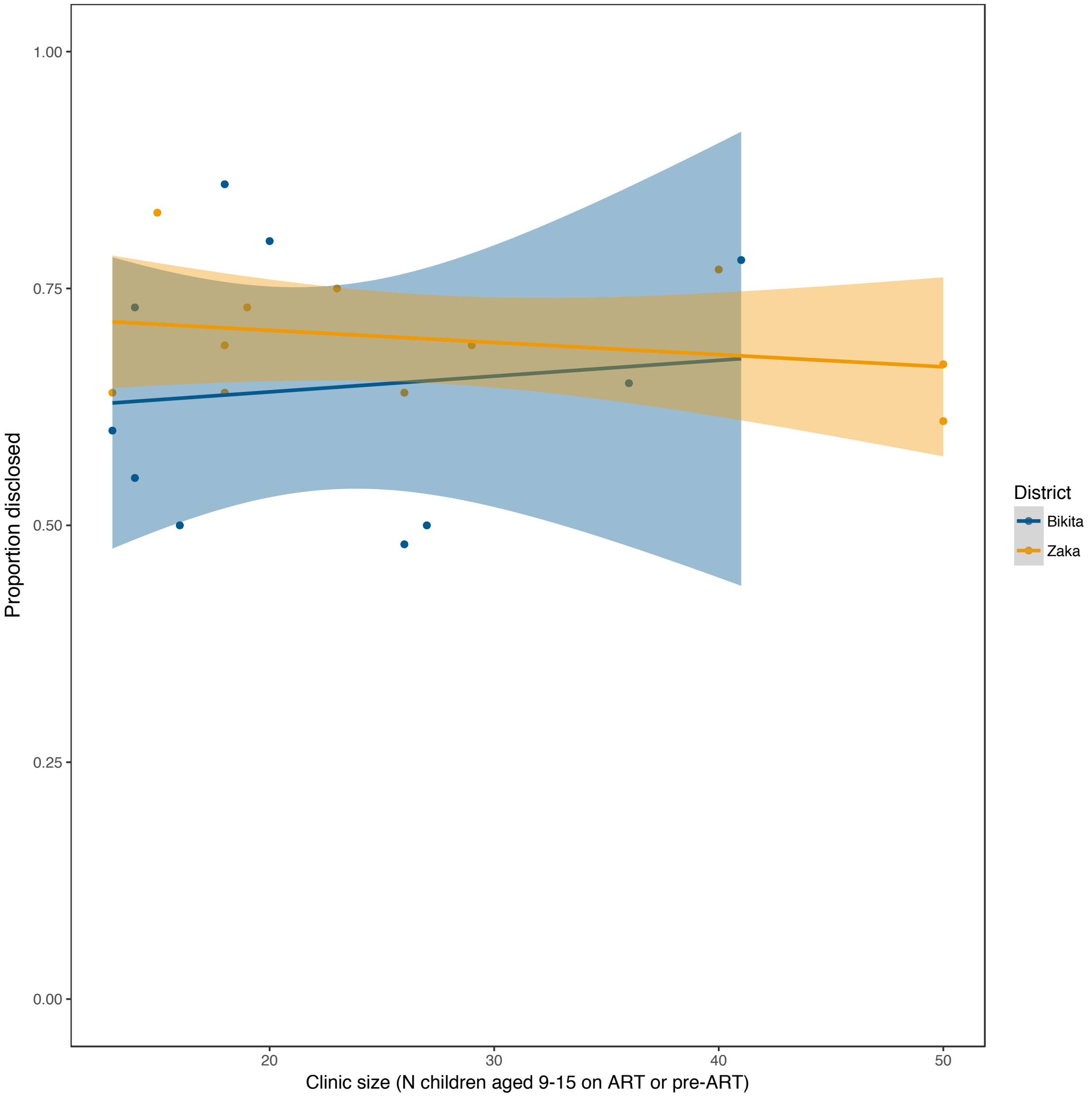

Figure B.1: **Disclosure by clinic size.** This figure shows how measured rates of disclosure vary by the size of the clinic. Clinic size is the number of children ages 9 to 15 on ART or pre-ART by clinic collected as part of the sampling frame. Rates of disclosure vary across clinics, but they do not seem to be associated with clinic population aged 9 to 15 on ART or pre-ART.

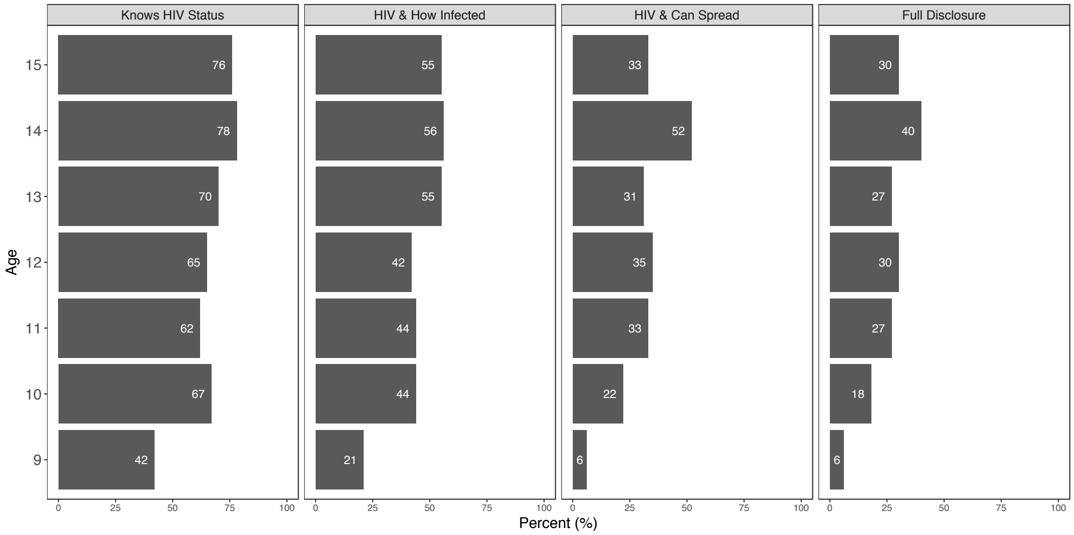

Figure B.2: **Percentage of children who know facts about their HIV status by age.** Sample includes all children whose caregivers were surveyed as part of the cross-sectional survey (Wave 1).

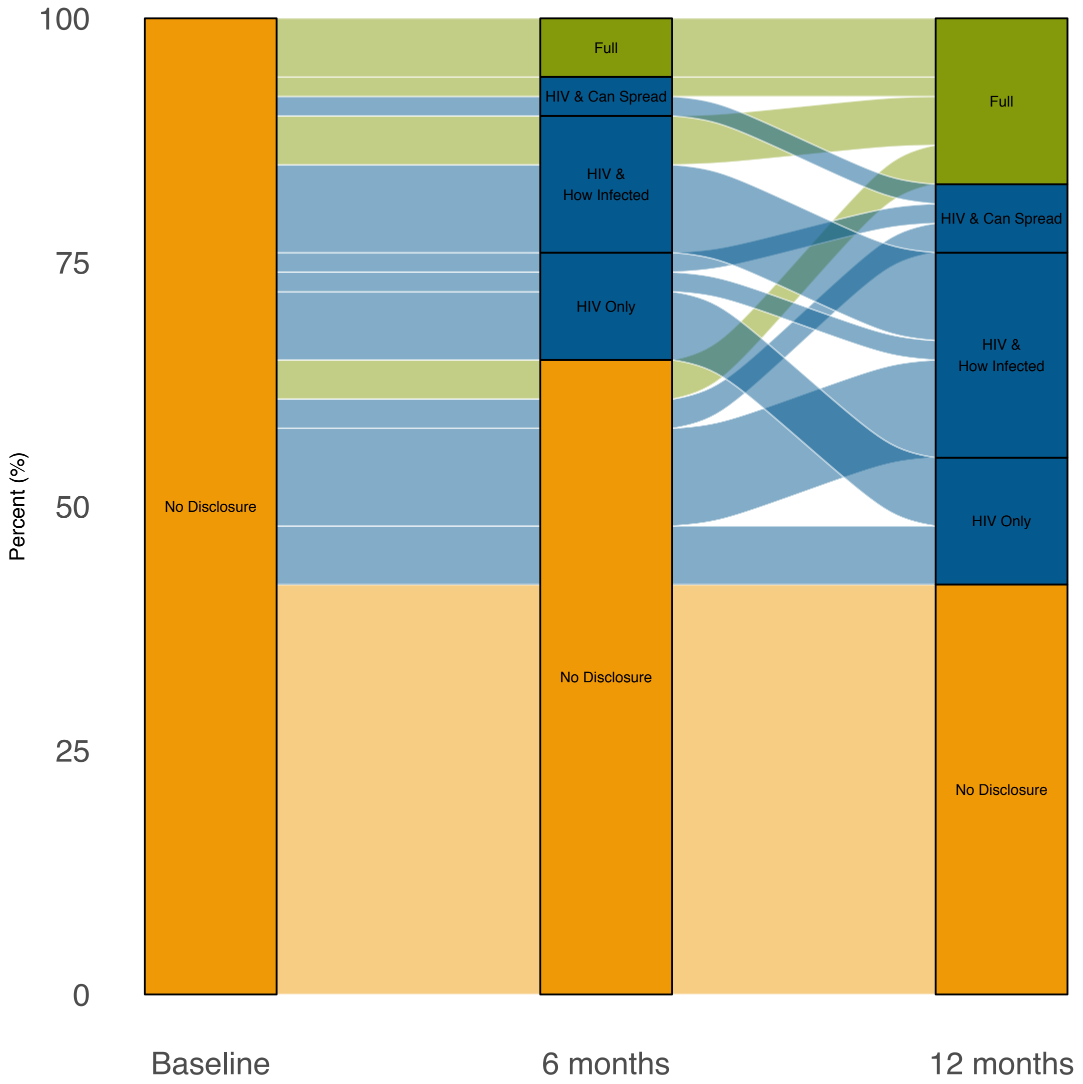

Figure B.3: **What children learned about their HIV status over time.** This figure shows a stacked bar chart at baseline (Wave 1), 6 months (Wave 2), and 12 months (Wave 3) of the proportion of children in the prospective cohort who knew certain elements of their HIV status. Fully disclosed children ("Full", indicated in green) knew that they have an illness called HIV, how they were infected, and that they can spread the virus to others. Partially disclosed children (indicated in blue) knew some but not all of these details. Children who do not know that they have an illness called HIV are labeled as "No Disclosure" (indicated in orange).

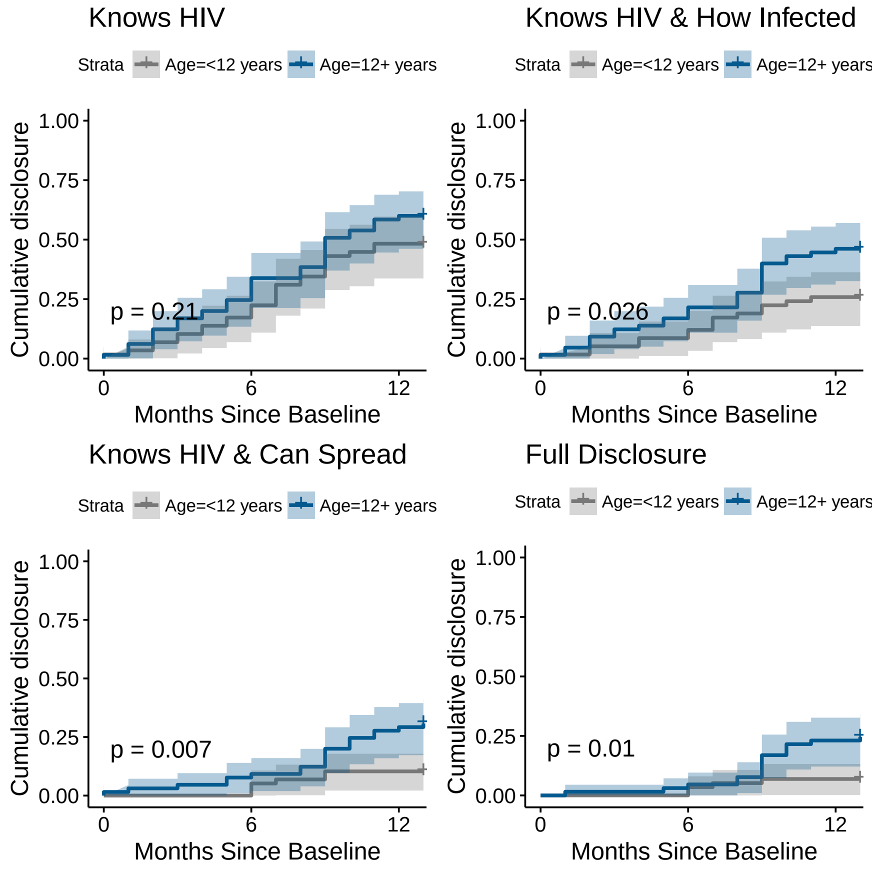

Figure B.4: **Rates of learning facts about HIV status among the prospective cohort disaggregated by age.** This figure shows the cumulative incidence of disclosure to children in the prospective cohort by age (under 12 years vs. 12 years and older). Each period represents 1 month from the baseline survey (Wave 1).

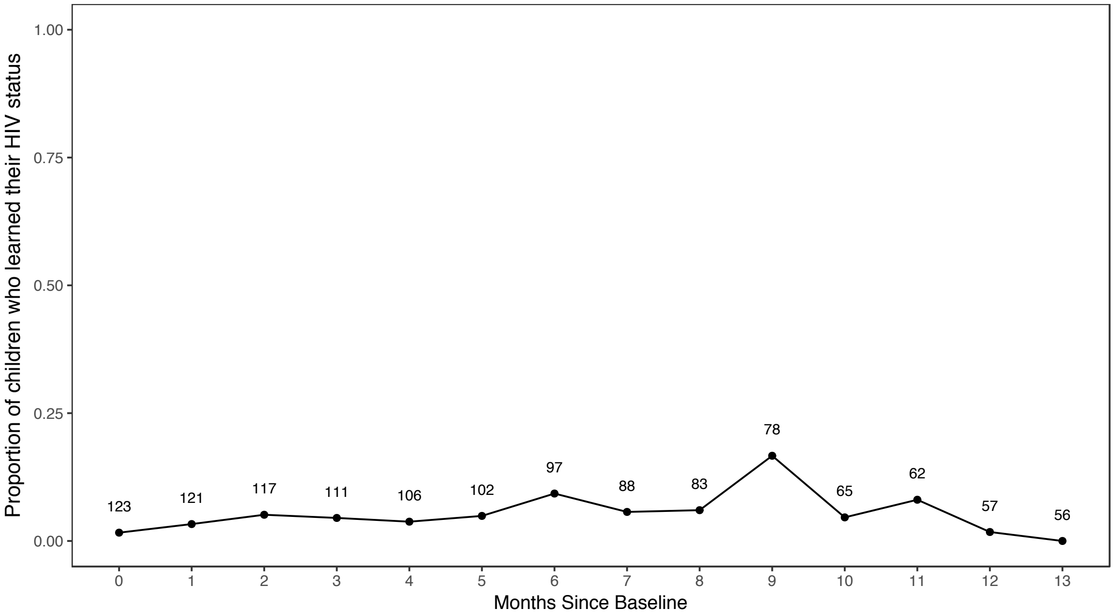

Figure B.5: **Monthly disclosure rate by months since baseline.** This figure shows the proportion of children in the prospective cohort who learned their HIV status each month since the baseline (Wave 1) among non-disclosed children, i.e. the children at risk for disclosure indicated by the number above each dot.

Table B.1: Caregiver relationship to child in sample

Caregiver relationship to child	N	%
Biological parent	169	45.40
Grandparent	108	29.00
Aunt or uncle	64	17.20
Other relative	13	3.50
Adopted, foster, or stepparent	7	1.90
Sibling	7	1.90
Other	3	0.80
Son or daughter (in law)	1	0.30

Note. Information for all caregivers surveyed at Wave 1 (N=372).

Table B.2: Disclosure status by wave

	Cross-Sectional Sample		Prospective Cohort (N=123)			
	Wave 1 (Baseline) N=372		Wave 2 (6 Months) N=112		Wave 3 (12 Months) N=117	
Child knows	%	(95% CI)	%	(95% CI)	%	(95% CI)
a. He/she has a medical condition requiring ongoing treatment	91.9	(88.7, 94.3)	90.2	(83.3, 94.4)	96.6	(91.5, 98.7)
b. Medical condition is called HIV	66.9	(62.0, 71.5)	36.6	(28.3, 45.8)	58.1	(49.1, 66.7)
c. How he/she aquired HIV	46.8	(41.8, 51.9)	21.4	(14.8, 29.9)	38.5	(30.1, 47.5)
d. He/she can spread the disease to others	32.5	(28.0, 37.4)	9.8	(5.6, 16.7)	23.1	(16.4, 31.5)
Full disclosure (all three of b-d)	26.9	(22.6, 31.6)	6.2	(3.1, 12.3)	17.1	(11.3, 24.9)
No disclosure (none of b-d)	33.1	(28.5, 38.0)	63.4	(54.2, 71.7)	41.9	(33.3, 50.9)
Partial disclosure (at least one of b-d)	40.1	(35.2, 45.1)	30.4	(22.6, 39.4)	41.0	(32.5, 50.1)

Table B.3: Worries about disclosure among non-disclosed caregivers at 12 months (N=49)

Caregiver was somewhat or very worried that telling the child will:	(%)
- just create more problems.	57.4
- make [him/her] too sad.	57.4
- lead [him/her] to ask more tough questions.	55.3
- make [him/her] think less of [him/her]self.	51.1
- make [him/her] less hopeful about the future.	51.1
- lead [him/her] to experience stigma in the community.	47.9
- lead [him/her] to have difficulty in school.	46.8
- actually bring [him/her] harm.	45.7
- cause me to feel too many bad emotions.	42.6
- cause [him/her] to struggle more every day with normal tasks.	40.4
- bring more bad things than good things.	38.3
- make [his/her] condition get worse.	34.0
- lead [him/her] to reject me.	23.4
- make [him/her] stop taking medication.	23.4
- put a strain on our family.	21.3

Note. Caregivers responded to each item on a 4-point scale as follows: (0) "not at all worried", (1) "not very worried", (2) "somewhat worried", and (3) "very worried." Percentages in the table represent those who responded that they were somewhat or very worried.

C Assessment of Disclosure Rates from the Literature

To place our results in context, we identified several systematic reviews of pediatric HIV disclosure studies [3, 4, 5, 6, 7, 8, 9], conducted a supplemental search of the peer reviewed literature, and extracted 51 reported estimates of pediatric HIV disclosure prevalence from the collection of reviews and single studies. When possible, we also derived estimates of HIV disclosure for specific age groups using information reported in the articles (21 additional estimates).

We plot all reported and derived estimates in Figure C.1. A black dot indicates that a study used probability sampling, and an unfilled grey circle indicates that the estimate was based on a convenience sample. Table C.1 displays the reported and derived estimates from each included study.

All of the studies in this collection – 52 in total with our study included – defined disclosure as the child knowing they have a virus/illness called HIV. Only 2/52 studies calculated the sample prevalence of disclosure based on child-report [10, 11]. The rest relied on caregiver-report (43 studies), medical records (3 studies), or did not specify the source (4 studies).

1 of the 2 studies that interviewed children to determine disclosure status, Turissini, et al. (2013), documented both caregiver-reported (27 of 270; 10.2%) and child-reported (20 of 266; 7.5%) values. The difference in disclosure prevalence between informants is negligible – 2.7 percentage points. We used the caregiver-reported value in Figure 4 and Figure C.1.

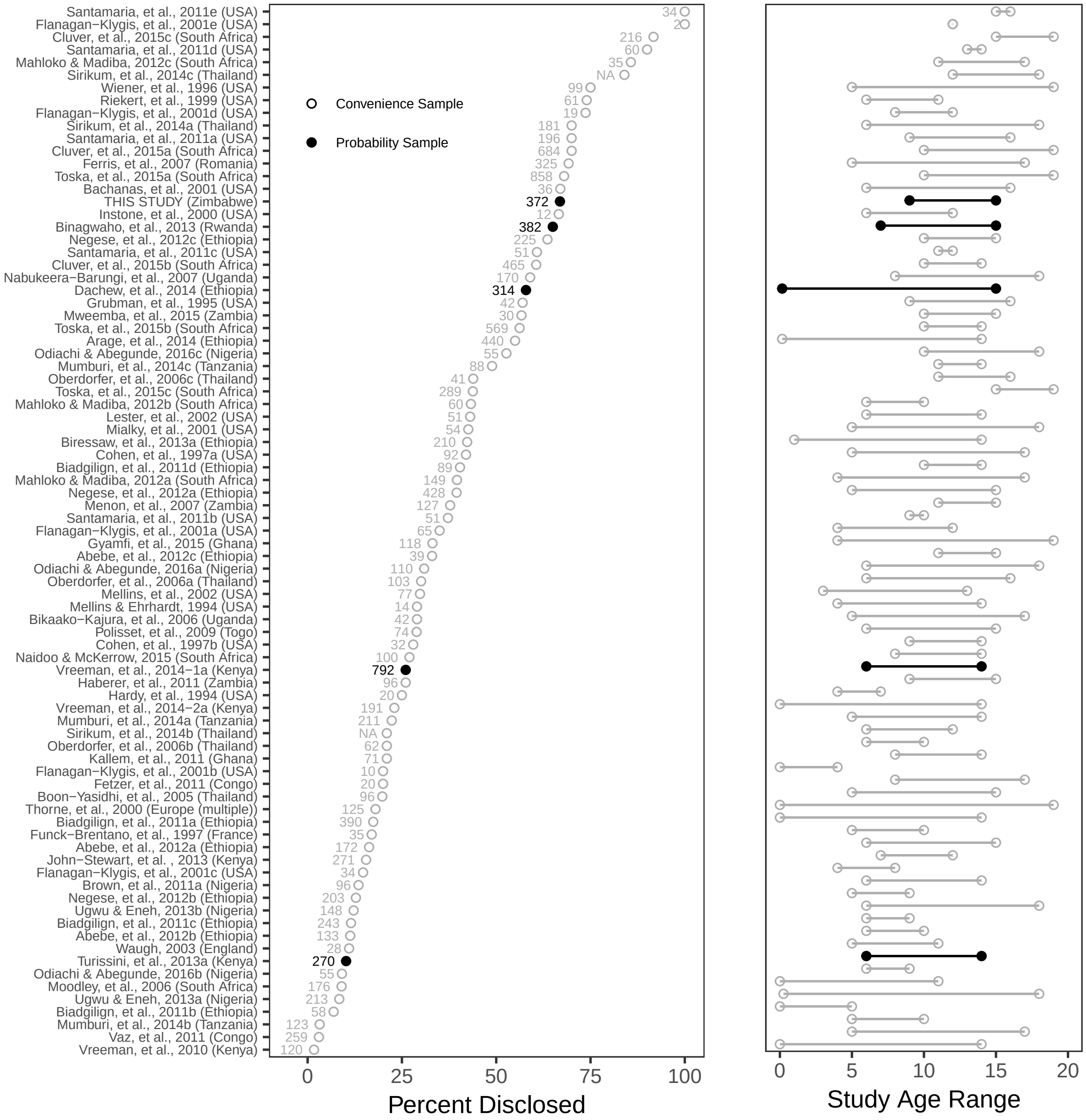

Figure C.1: **Disclosure of HIV status of children across studies.** This figure displays the rates of pediatric HIV disclosure (i.e. the child knows that the name of the illness they have is HIV) reported in 52 studies (including our study) plus derived estimates, the location and sample size of each study, whether the study used probability sampling methods (black filled dots), and the age range of the children. We compiled the data for this figure from recent systematic reviews [3, 4, 5, 6, 7, 8, 9] and our own supplemental search of the literature. Reported estimates either have the letter "a" or no letter appended to the study year; derived estimates have a letter other than "a" appended to the study year.

Table C.1: Studies included in literature figure

		Child's Age Range				
Study	Country	Min	Max	Disclosed (%)	Sample Size	Source
Flanagan-Klygis, et al. (2001e) [12]	USA	12	12	100.00	24	reported
Santamaria, et al. (2011e) [13]	USA	15	16	100.00	43	derived
Cluver, et al. (2015c) [14]	South Africa	15	19	91.70	30	reported
Santamaria, et al. (2011d) [13]	USA	13	14	90.00	62	derived
Mahloko & Madiba (2012c) [15]	South Africa	11	17	85.70	44	derived
Sirikum, et al. (2014c) [16]	Thailand	12	18	84.00	78	reported
Wiener, et al. (1996) [17]	USA	5	19	75.00	77	reported
Riekert, et al. (1999) [18]	USA	6	11	74.00	63	reported
Flanagan-Klygis, et al. (2001d) [12]	USA	8	12	73.70	21	reported
Cluver, et al. (2015a) [14]	South Africa	10	19	70.00	66	reported
Santamaria, et al. (2011a) [13]	USA	9	16	70.00	23	reported
Sirikum, et al. (2014a) [16]	Thailand	6	18	70.00	20	reported
Ferris, et al. (2007) [19]	Romania	5	17	69.20	42	reported
Toska, et al. (2015a) [20]	South Africa	10	19	68.00	71	reported
Bachanas, et al. (2001) [21]	USA	6	16	67.00	45	reported
THIS STUDY	Zimbabwe	9	15	66.90	46	reported
Instone, et al. (2000) [22]	USA	6	12	66.60	6	reported
Binagwaho, et al. (2013) [23]	Rwanda	7	15	65.00	47	reported
Negese, et al. (2012c) [24]	Ethiopia	10	15	63.60	31	derived
Santamaria, et al. (2011c) [13]	USA	11	12	60.80	57	derived
Cluver, et al. (2015b) [14]	South Africa	10	14	60.60	55	reported
Nabukeera-Barungi, et al. (2007) [25]	Uganda	8	18	59.00	17	reported
Dachew, et al. (2014) [26]	Ethiopia	<1	15	57.90	40	reported
Grubman, et al. (1995) [27]	USA	9	16	57.00	52	reported
Mweemba, et al. (2015) [28]	Zambia	10	15	56.70	38	reported
Toska, et al. (2015b) [20]	South Africa	10	14	56.20	60	reported
Arage, et al. (2014) [29]	Ethiopia	<1	14	55.00	54	reported
Odiachi & Abegunde (2016c) [30]	Nigeria	10	18	52.70	59	derived
Mumburi, et al. (2014c) [31]	Tanzania	11	14	48.90	73	derived
Oberdorfer, et al. (2006c) [32]	Thailand	11	16	43.90	51	derived
Toska, et al. (2015c) [20]	South Africa	15	19	43.80	37	reported
Mahloko & Madiba (2012b) [15]	South Africa	6	10	43.30	62	derived
Lester, et al. (2002) [33]	USA	6	14	43.10	57	reported
Mialky, et al. (2001) [34]	USA	5	18	42.60	58	reported
Biressaw, et al. (2013a) [35]	Ethiopia	1	14	42.30	27	reported
Cohen, et al. (1997a) [36]	USA	5	17	42.00	75	reported
Biadgilign, et al. (2011d) [37]	Ethiopia	10	14	40.40	74	derived
Mahloko & Madiba (2012a) [15]	South Africa	4	17	39.60	15	reported
Negese, et al. (2012a) [24]	Ethiopia	5	15	39.50	53	reported
Menon, et al. (2007) [38]	Zambia	11	15	37.80	10	reported
Santamaria, et al. (2011b) [13]	USA	9	10	37.20	57	derived
Flanagan-Klygis, et al. (2001a) [12]	USA	4	12	35.00	65	reported
Gyamfi, et al. (2015) [39]	Ghana	4	19	33.10	5	reported
Abebe, et al. (2012c) [40]	Ethiopia	11	15	33.00	48	derived
Odiachi & Abegunde (2016a) [30]	Nigeria	6	18	30.90	4	reported
Oberdorfer, et al. (2006a) [32]	Thailand	6	16	30.10	3	reported
Mellins, et al. (2002) [41]	USA	3	13	29.80	69	reported
Bikaako-Kajura, et al. (2006) [42]	Uganda	5	17	29.00	52	reported
Mellins & Ehrhardt (1994) [43]	USA	4	14	29.00	12	reported
Polisset, et al. (2009) [44]	Togo	6	15	28.90	68	reported
Cohen, et al. (1997b) [36]	USA	9	14	28.00	41	derived
Naidoo & McKerrow (2015) [45]	South Africa	8	14	27.00	2	reported
Haberer, et al. (2011) [46]	Zambia	9	15	26.00	76	reported
Vreeman, et al. (2014-1a) [47]	Kenya	6	14	26.00	70	reported
Hardy, et al. (1994) [48]	USA	4	7	25.00	25	reported
Vreeman, et al. (2014-2a) [11]	Kenya	<1	14	23.00	22	reported
Mumburi, et al. (2014a) [31]	Tanzania	5	14	22.30	28	reported
Kallem, et al. (2011) [49]	Ghana	8	14	21.00	67	reported
Oberdorfer, et al. (2006b) [32]	Thailand	6	10	21.00	64	derived
Sirikum, et al. (2014b) [16]	Thailand	6	12	21.00	78	reported
Fetzer, et al. (2011) [50]	Congo	8	17	20.00	25	reported
Flanagan-Klygis, et al. (2001b) [12]	USA	<1	4	20.00	1	reported
Boon-Yasidhi, et al. (2005) [51]	Thailand	5	15	19.80	76	reported
Thorne, et al. (2000) [52]	Europe (multiple)	<1	19	18.00	9	reported
Biadgilign, et al. (2011a) [37]	Ethiopia	<1	14	17.40	49	reported
Funck-Brentano, et al. (1997) [53]	France	5	10	17.00	44	reported
Abebe, et al. (2012a) [40]	Ethiopia	6	15	16.30	18	reported
John-Stewart, et al. (2013) [54]	Kenya	7	12	15.50	35	reported
Flanagan-Klygis, et al. (2001c) [12]	USA	4	8	14.70	43	reported
Brown, et al. (2011a) [55]	Nigeria	6	14	13.50	76	reported
Negese, et al. (2012b) [24]	Ethiopia	5	9	12.80	26	derived
Ugwu & Eneh (2013b) [56]	Nigeria	6	18	12.20	14	derived
Biadgilign, et al. (2011c) [37]	Ethiopia	6	9	11.50	32	derived
Abebe, et al. (2012b) [40]	Ethiopia	6	10	11.30	11	derived
Waugh (2003) [57]	England	5	11	11.00	36	reported
Turissini, et al. (2013a) [10]	Kenya	6	14	10.20	34	reported
Odiachi & Abegunde (2016b) [30]	Nigeria	6	9	9.10	59	derived
Moodley, et al. (2006) [58]	South Africa	<1	11	9.00	19	reported
Ugwu & Eneh (2013a) [56]	Nigeria	<1	18	8.40	29	reported
Biadgilign, et al. (2011b) [37]	Ethiopia	<1	5	6.90	61	derived
Mumburi, et al. (2014b) [31]	Tanzania	5	10	3.20	8	derived
Vaz, et al. (2011) [59]	Congo	5	17	3.00	33	reported
Vreeman, et al. (2010) [60]	Kenya	<1	14	1.70	7	reported

Note. This table shows estimates of pediatric HIV disclosure (i.e. the child knows that the name of the illness they have is HIV) from published systematic reviews (reported) and estimates derived from these studies not published in the systematic reviews (derived). Derived estimates were calculated directly from each published study if the study reported disclosure rates among subsets of their study population.

References

[1] J. Larmarange, V. Bendaud, Hiv estimates at second subnational level from national population-based surveys, AIDS 28 (Suppl 4) (2014) S469–S476.

[2] UNAIDS, Zimbabwe developing subnational estimates of HIV prevalence and the number of people living with HIV (2014).

[3] B. J. Krauss, S. Letteney, C. N. Okoro, Why tell children: A synthesis of the global literature on reasons for disclosing or not disclosing an HIV diagnosis to children 12 and under, Front Public Health 4.

[4] R. C. Vreeman, A. M. Gramelspacher, P. O. Gisore, M. L. Scanlon, W. M. Nyandiko, Disclosure of HIV status to children in resource-limited settings: a systematic review, Journal of the International AIDS Society 16 (1).

[5] M. C. Pinzón-Iregui, C. M. Beck-Sagué, R. M. Malow, Disclosure of their HIV status to infected children: A review of the literature, J Trop Pediatr 59 (2) (2013) 84–89.

[6] J. Nichols, A. Steinmetz, P. Elijah, Impact of HIV-status disclosure on adherence to antiretroviral therapy among HIV-infected children in resource-limited aettings: A systematic review, AIDS Behav 21 (1) (2017) 1–11. doi:10.1007/s10461-016-1481-z.

[7] L. Wiener, C. A. Mellins, S. Marhefka, H. B. Battles, Disclosure of an HIV diagnosis to children: history, current research, and future directions, Journal of Developmental & Behavioral Pediatrics 28 (2) (2007) 155–166.

[8] C. Britto, K. Mehta, R. Thomas, A. Shet, Prevalence and correlates of HIV disclosure among children and adolescents in low- and middle-income countries: A

systematic review, J Dev Behav Pediatr 37 (6) (2016) 496–505.

[9] O. Aderomilehin, A. Hanciles-Amu, O. O. Ozoya, Perspectives and practice of HIV disclosure to children and adolescents by health-care providers and caregivers in sub-saharan africa: A systematic review, Front Public Health 4 (166).

[10] M. L. Turissini, W. M. Nyandiko, S. O. Ayaya, I. Marete, A. Mwangi, V. Chemboi, L. Warui, R. C. Vreeman, The prevalence of disclosure of HIV status to HIV-infected children in Western Kenya, Journal of the Pediatric Infectious Diseases Society 2 (2) (2013) 136–143. doi:10.1093/jpids/pit024.

[11] R. C. Vreeman, M. L. Scanlon, A. Mwangi, M. Turissini, S. O. Ayaya, C. Tenge, W. M. Nyandiko, A Cross-Sectional Study of Disclosure of HIV Status to Children and Adolescents in Western Kenya, PLoS ONE 9 (1) (2014) e86616. doi:10.1371/journal.pone.0086616.

[12] E. Flanagan-Klygis, L. Friedman Ross, J. Lantos, J. Frader, R. Yogev, Disclosing the diagnosis of HIV in pediatrics, The Journal of Clinical Ethics 12 (2) (2001) 150–157.

[13] E. Santamaria, Karina, C. Dolezal, S. Marhefka, L., S. Hoffman, Y. Ahmed, K. Elkington, C. Mellins, A., Psychosocial Implications of HIV Serostatus Disclosure to Youth with Perinatally Acquired HIV, AIDS Patient Care & STDs 25 (4) (2011) 257–264.

[14] L. D. Cluver, R. J. Hodes, E. Toska, K. K. Kidia, F. M. Orkin, L. Sherr, F. Meinck, ‘HIV is like a tsotsi. ARVs are your guns’: associations between HIV-disclosure and adherence to antiretroviral treatment among adolescents in South Africa, AIDS 29 (2015) S57–S65.

[15] J. M. Mahloko, S. Madiba, Disclosing HIV diagnosis to children in Odi district, South Africa : reasons for disclosure and non-disclosure, Afr J Prm Health Care Fam Med 4 (1).

[16] C. Sirikum, J. Sophonphan, T. Chuanjaroen, S. Lakonphon, A. Srimuan, P. Chusut, T. C. Do, W. Prasitsuebsai, T. Puthanakit, J. Ananworanich, T. Bunupuradah, on behalf of HIV-NAT 015 study team, HIV disclosure and its effect on treatment outcomes in perinatal HIV-infected Thai children, AIDS Care 26 (9) (2014) 1144–1149.

[17] L. S. Wiener, H. B. Battles, N. Heilman, C. K. Sigelman, P. A. Pizzo, Factors associated with disclosure of diagnosis to children with HIV/AIDS., Pediatric AIDS and HIV infection 7 (5) (1996) 310–324.

[18] K. A. Riekert, L. Wiener, H. Battles, Prediction of psychological disress in school-age children with HIV, Children's Health Care 28 (3) (1999) 201–220.

[19] M. Ferris, K. Burau, A. Schweitzer, S. Mihale, N. Murray, A. Preda, M. Ross, M. Kline, The influence of disclosure of HIV diagnosis on time to disease progression in a cohort of Romanian children and teens, AIDS Care 19 (9) (2007) 1088–1094.

[20] E. Toska, L. D. Cluver, R. Hodes, K. K. Kidia, Sex and secrecy: How HIV-status disclosure affects safe sex among HIV-positive adolescents, AIDS Care 27 (sup1) (2015) 47–58. doi:10.1080/09540121.2015.1071775.

[21] P. j. Bachanas, K. A. Kullgren, K. S. Schwartz, B. Lanier, J. S. McDaniel, J. Smith, S. Nesheim, Predictors of psychological adjustment in school age children infected with HIV, Journal of Pediatric Psychology 26 (6) (2001) 343–352.

[22] S. L. Instone, Perceptions of children with HIV infection when not told for so long: Implications for diagnosis disclosure, Journal of Pediatric Health Care 14 (5) (2000) 235–243.

[23] A. Binagwaho, I. Murekatete, A. Rukundo, P. Mugwaneza, R. Hinda, A. Lyambabaje, J. Bizimana, C. Wagner, G. Muriisa, J. Muita, V. Mutabaxi, J. P. Nyemazi, S. Nsanzimana, C. Karema, Factors associated with disclosure of HIV status among HIV positive children in Rwanda, Rwanda Medical Journal 69 (3).

[24] D. Negese, K. Addis, A. Awoke, Z. Birhanu, D. Muluye, S. Yifru, B. Megabiaw, HIV-Positive Status Disclosure and Associated Factors among Children in North Gondar, Northwest Ethiopia, International Scholarly Research Notices 2012 (2012) e485720.

[25] N. Nabukeera-Barungi, I. Kalyesubula, A. Kekitiinwa, Byakika-Tusiime, P. Musoke, Adherence to antiretroviral therapy in children attending Mulago Hospital, Kampala, Annals of Tropical Paediatrics 27 (2) (2007) 123–131.

[26] B. A. Dachew, T. B. Tesfahunegn, A. M. Birhanu, Adherence to highly active antiretroviral therapy and associated factors among children at the University of Gondar Hospital and Gondar Poly Clinic, Northwest Ethiopia: a cross-sectional institutional based study, BMC Public Health 14 (875) (2014) 1–6.

[27] S. Grubman, E. Gross, N. Lerner-Weiss, M. Hernandez, G. D. McSherry, L. G. Hoyt, M. Boland, J. M. Oleske, Older children and adolescents living with perinatally acquired human immunodeficiency virus infection, Pediatrics 95 (5) (1995) 657–663.

[28] M. Mweemba, M. M. Musheke, C. Michelo, H. Halwiindi, O. Mweemba, J. M. Zulu, "When am I going to stop taking the drug?" Enablers, barriers and processes of

disclosure of HIV status by caregivers to adolescents in a rural district in Zambia, BMC Public Health 15 (1).

[29] G. Arage, G. A. Tessema, H. Kassa, Adherence to antiretroviral therapy and its associated factors among children at South Wollo Zone Hospitals, Northeast Ethiopia: a cross-sectional study, BMC Public Health 14 (1) (2014) 365.

[30] A. Odiachi, D. Abegunde, Prevalence and predictors of pediatric disclosure among HIV-infected Nigerian children on treatment, AIDS Care 28 (8) (2016) 1046–1051. doi:10.1080/09540121.2016.1147018.

[31] L. P. Mumburi, B. C. Hamel, R. N. Philemon, G. N. Kapanda, L. J. Msuya, Factors associated with HIV-status disclosure to HIV-infected children receiving care at Kilimanjaro Christian Medical Centre in Moshi, Tanzania, The Pan African Medical Journal 18.

[32] P. Oberdorfer, T. Puthanakit, O. Louthrenoo, C. Charnsil, V. Sirisanthana, T. Sirisanthana, Disclosure of HIV/AIDS diagnosis to HIV-infected children in Thailand, Journal of Paediatrics and Child Health 42 (5) (2006) 283–288.

[33] P. Lester, M. Chesney, M. Cooke, P. Whalley, B. Perez, A. Petru, A. Dorenbaum, D. Wara, Diagnostic disclosure to HIV-infected children: how parents decide when and what to tell, Clinical child psychology and psychiatry 7 (1) (2002) 85–99.

[34] E. Mialky, J. Vagnoni, R. Rutstein, School-age children with perinatally acquired HIV infection: medical and psychosocial issues in a Philadelphia cohort, AIDS patient care and STDs 15 (11) (2001) 575–579.

[35] S. Biressaw, W. E. Abegaz, M. Abebe, W. A. Taye, M. Belay, Adherence to Antiretroviral Therapy and associated factors among HIV infected children in

Ethiopia: unannounced home-based pill count versus caregivers' report, BMC pediatrics 13 (1) (2013) 132.

[36] J. Cohen, C. Reddington, D. Jacobs, R. Meade, D. Picard, K. Singleton, D. Smith, M. B. Caldwell, A. DeMaria, H.-W. Hsu, others, School-related issues among HIV-infected children, Pediatrics 100 (1) (1997) e8–e8.

[37] S. Biadgilign, A. Deribew, A. Amberbir, H. R. Escudero, K. Deribe, Factors Associated with HIV/AIDS Diagnostic Disclosure to HIV Infected Children Receiving HAART: A Multi-Center Study in Addis Ababa, Ethiopia, PLoS ONE 6 (3) (2011) e17572.

[38] A. Menon, C. Glazebrook, N. Campain, M. Ngoma, Mental Health and Disclosure of HIV Status in Zambian Adolescents With HIV Infection: Implications for Peer-Support Programs, JAIDS Journal of Acquired Immune Deficiency Syndromes 46 (3) (2007) 349–354.

[39] E. Gyamfi, P. Okyere, E. Appiah-Brempong, R. O. Adjei, K. A. Mensah, Benefits of Disclosure of HIV Status to Infected Children and Adolescents: Perceptions of Caregivers and Health Care Providers, Journal of the Association of Nurses in AIDS Care 26 (6) (2015) 770–780.

[40] W. Abebe, S. Teferra, Disclosure of diagnosis by parents and caregivers to children infected with HIV: Prevalence associated factors and perceived barriers in Addis Ababa, Ethiopia, AIDS Care 24 (9) (2012) 1097–1102. doi:10.1080/09540121.2012.656565.

[41] C. A. Mellins, E. Brackis-Cott, C. Dolezal, A. Richards, S. W. Nicholas, E. J. Abrams, Patterns of HIV status disclosure to perinatally HIV-infected children

and subsequent mental health outcomes, Clinical Child Psychology and Psychiatry 7 (1) (2002) 101–114.

[42] W. Bikaako-Kajura, E. Luyirika, D. W. Purcell, J. Downing, F. Kaharuza, J. Mermin, S. Malamba, R. Bunnell, Disclosure of HIV status and adherence to daily drug regimens among HIV-infected children in Uganda, AIDS and Behavior 10 (S1) (2006) 85–93.

[43] C. A. Mellins, A. A. Ehrhardt, Families affected by pediatric acquired immunodeficiency syndrome: Sources of stress and coping, Developmental and Behavioral Pediatrics 15 (3).

[44] J. Polisset, F. Ametonou, E. Arrive, A. Aho, F. Perez, Correlates of Adherence to Antiretroviral Therapy in HIV-Infected Children in Lomé, Togo, West Africa, AIDS and Behavior 13 (1) (2009) 23–32.

[45] G. D. Naidoo, N. H. McKerrow, Current practices around HIV disclosure to children on highly active antiretroviral therapy, South African Journal of Child Health 9 (3) (2015) 85.

[46] J. E. Haberer, A. Cook, A. S. Walker, M. Ngambi, A. Ferrier, V. Mulenga, C. Kityo, M. Thomason, D. Kabamba, C. Chintu, D. M. Gibb, D. R. Bangsberg, Excellent Adherence to Antiretrovirals in HIV+ Zambian Children Is Compromised by Disrupted Routine, HIV Nondisclosure, and Paradoxical Income Effects, PLoS ONE 6 (4) (2011) e18505.

[47] R. C. Vreeman, W. M. Nyandiko, H. Liu, W. Tu, M. L. Scanlon, J. E. Slaven, S. O. Ayaya, T. S. Inui, Measuring adherence to antiretroviral therapy in children and adolescents in western Kenya, Journal of the International AIDS Society 17 (1).

[48] M. S. Hardy, F. D. Armstrong, D. K. Routh, J. Albrecht, J. Davis, Coping and communication among parents and children with human immunodeficiency virus and cancer, Developmental and Behavioral Pediatrics 15 (3).

[49] S. Kallem, L. Renner, M. Ghebremichael, E. Paintsil, Prevalence and Pattern of Disclosure of HIV Status in HIV-Infected Children in Ghana, AIDS and Behavior 15 (6) (2011) 1121–7.

[50] B. C. Fetzer, B. Mupenda, J. Lusiama, F. Kitetele, C. Golin, F. Behets, Barriers to and facilitators of adherence to pediatric antiretroviral therapy in a sub-Saharan setting: insights from a qualitative study, AIDS patient care and STDs 25 (10) (2011) 611–621.

[51] V. Boon-Yasidhi, U. Kottapat, Y. Durier, N. Plipat, W. Phongsamart, K. Chokephaibulkit, N. Vanprapar, Diagnosis disclosure in HIV-infected Thai children, J Med Assoc Thai 88 (Suppl 8) (2005) S100–5.

[52] C. Thorne, M.-L. Newell, C. Pckham, Disclosure of diagnosis and planning for the future in HIV-affected families in Europe, Child: Care, Health and Development 26 (1) (2000) 29–40.

[53] Funck-Brentano I, Costagliola D, Seibel N, Straub E, Tardieu M, Blanche S, Patterns of disclosure and perceptions of the human immunodeficiency virus in infected elementary school—age children, Archives of Pediatrics & Adolescent Medicine 151 (10) (1997) 978–985.

[54] G. John-Stewart, C., G. Wariua, K. Beima-Sofie, M., B. Richardson, A., C. Farquhar, E. Maleche-Obimbo, D. Mbori-Ngacha, D. Wamalwa, Prevalence, perceptions, and correlates of pediatric HIV disclosure in an HIV treatment program in Kenya, AIDS Care 25 (9) (2013) 1067–1076.

[55] B. Brown, J., R. Oladokun, E., K. Osinusi, S. Ochigbo, I. Adewole, F., P. Kanki, Disclosure of HIV status to infected children in a Nigerian HIV Care Programme, AIDS Care 23 (9) (2011) 1053–1058.

[56] R. Ugwu, A. Eneh, Factors influencing adherence to paediatric antiretroviral therapy in Portharcourt, South- South Nigeria, Pan African Medical Journal 16.

[57] S. Waugh, Parental views on disclosure of diagnosis to their HIV-positive children, AIDS Care 15 (2) (2003) 169–176.

[58] K. Moodley, L. Myer, D. Michaels, M. Cotton, Paediatric HIV disclosure in South Africa-caregivers' perspectives on discussing HIV with infected children, South African Medical Journal 96 (3) (2006) 201–204.

[59] L. M. E. Vaz, S. Maman, E. Eng, O. A. Barbarin, T. Tshikandu, F. Behets, Patterns of Disclosure of HIV- Status to Infected Children in a Sub-Saharan African Setting, Journal of developmental and behavioral pediatrics : JDBP 32 (4) (2011) 307–315.

[60] R. C. Vreeman, W. M. Nyandiko, S. O. Ayaya, E. G. Walumbe, D. G. Marrero, T. S. Inui, The Perceived Impact of Disclosure of Pediatric HIV Status on Pediatric Antiretroviral Therapy Adherence, Child Well-Being, and Social Relationships in a Resource-Limited Setting, AIDS Patient Care and STDs 24 (10) (2010) 639–649.
